# Serological Evidence of Human Orthohantavirus Infections in Barbados, 2008 to 2016

**DOI:** 10.3390/pathogens10050571

**Published:** 2021-05-08

**Authors:** Kirk Osmond Douglas, Thelma Alafia Samuels, Rommel Iheozor-Ejiofor, Olli Vapalahti, Tarja Sironen, Marquita Gittens-St. Hilaire

**Affiliations:** 1 Centre for Biosecurity Studies, University of the West Indies, Cave Hill, St. Michael BB11000, Barbados; 2 Epidemiology Research Unit, Caribbean Institute for Health Research (CAIHR), The University of the West Indies, Mona, Kingston 7, Jamaica; alafiasam@gmail.com; 3 Department of Virology, Faculty of Medicine, Medicum, University of Helsinki, Haartmaninkatu 3, 00290 Helsinki, Finland; rommel.iheozor-ejiofor@helsinki.fi (R.I.-E.); olli.vapalahti@helsinki.fi (O.V.); tarja.sironen@helsinki.fi (T.S.); 4 Best-dos Santos Public Health Laboratory, Enmore #6, Lower Collymore Rock, St. Michael BB11155, Barbados; marquita.gittens@cavehill.uwi.edu; 5 Faculty of Medical Sciences, University of the West Indies, Cave Hill, St. Michael BB11000, Barbados

**Keywords:** HFRS, HPS, VHF, orthohantavirus, dengue, infectious disease, Caribbean, Americas, biosecurity

## Abstract

Background: Hantavirus pulmonary syndrome (HPS) is well-known in South and North America; however, not enough data exist for the Caribbean. The first report of clinical orthohantavirus infection was obtained in Barbados, but no other evidence of clinical orthohantavirus infections among adults in the Caribbean has been documented. Methods: Using enzyme linked immunosorbent assay (ELISA) tests followed by confirmatory testing with immunofluorescent assays (IFA), immunochromatographic (ICG) tests, and pseudotype focus reduction neutralization tests (pFRNT), we retrospectively and prospectively detected orthohantavirus-specific antibodies among patients with febrile illness in Barbados. Results: The orthohantavirus prevalence rate varied from 5.8 to 102.6 cases per 100,000 persons among febrile patients who sought medical attention annually between 2008 and 2016. Two major orthohantavirus epidemics occurred in Barbados during 2010 and 2016. Peak orthohantavis infections were observed observed during the rainy season (August) and prevalence rates were significantly higher in females than males and in patients from urban parishes than rural parishes. Conclusions: Orthohantavirus infections are still occurring in Barbados and in some patients along with multiple pathogen infections (CHIKV, ZIKV, DENV and *Leptospira*). Orthohantavirus infections are more prevalent during periods of high rainfall (rainy season) with peak transmission in August; females are more likely to be infected than males and infections are more likely among patients from urban rather than rural parishes in Barbados.

## 1. Introduction

Orthohantaviruses are single-stranded negative-sense RNA viruses approximately 120 to 160 nm in diameter from the Hantaviridae virus family that are maintained in rodent reservoirs [1,2,3]. Orthohantaviruses can be separated into two geographical groups, Old World (Seoul, Dobrava, Puumala and Hantaan) and New World (Prospect Hill, Andes, Sin Nombre, etc.) [1,2].

There are approximately 58 unique orthohantaviruses listed by the International Committee on Taxonomy of Viruses distributed globally, which are arranged in the Hantaviridae family and Orthohantavirus genus where in some cases different orthohantavirus strains are arranged in the same species, e.g., Dobrava virus (DOBV), Kurkino virus, Saaremaa virus, and Sochi virus are currently considered distinct viruses all belonging to the same species, *Dobrava-Belgrade orthohantavirus* [3,4,5]. Prospect Hill virus (PHV), the first known American orthohantavirus, was detected in humans in 1984 [6] and was followed by the 1993 outbreak of Sin Nombre virus (SNV) [7] in North America and the 1995 cases of Andes virus (ANDV) in South America [8,9]. The identification of novel orthohantaviruses and genotypes continues to occur due to enhanced research globally and especially in North and South America, where some 20 endemic and distinct viruses within 12 virus species have been identified [10].

Humans are incidental hosts of othohantaviruses and are typically infected via contaminated aerosolized secretions (feces, urine and saliva) of the reservoir animals. It is estimated that 150,000 to 200,000 annual orthohantavirus cases occur globally; however, this is likely to be an underestimate due to a lack of diagnostic testing and even awareness of this disease in some places [11]. Orthohantavirus infection can cause two main clinical diseases, namely haemorrhagic fever with renal syndrome (HFRS) and hantavirus pulmonary syndrome (HPS) or hantavirus cardiopulmonary syndrome (HCPS). Old-World hantaviruses are responsible for causing HFRS and a mild form of HFRS, nephropathica epidemica (NE), whereas New-World orthohantaviruses are responsible for HPS or HCPS. HFRS caused by HTNV can cause mortality rates up to 15% in Asian regions, while NE has a case fatality rate of 0.1 to 1% [12,13]. However, HPS is associated with mortality rates of 30 to 50% in North and South America [14,15,16]. Within the Caribbean context, other clinical presentations of orthohantavirus infections should be considered due to their similarity with other endemic infectious diseases caused by, for example, dengue virus (DENV), Zika virus (ZIKV) and Chikungunya virus (CHIKV) and *Leptospira* infections [17,18]. 

The first serological evidence of human orthohantavirus infections in the Caribbean involved the detection of anti-orthohantavirus antibodies in suspected leptospirosis patients in Barbados [19]. In this study, 12% of 60 patients presenting with febrile illness possessed orthohantavirus-specific immunoglobulin M (IgM) [19]. A later study of hospitalized children also demonstrated serological evidence of orthohantavirus infection [20]. However, the identity of the circulating orthohantavirus strain(s) and their source has remained unknown [6]. Evidence of human orthohantavirus infections, the presence of multiple rodent hosts in Venezuela [21,22,23,24], and a recent HPS outbreak in French Guiana in 2016 enhance the risk of new and more lethal orthohantavirus strains entering the Caribbean region via trade and travel [25].

Since the first orthohantavirus survey in Barbados, no other orthohantavirus serosurvey inclusive of adults has been conducted [19]. Given the known rodent fauna present in Barbados (*Rattus* spp. and *Mus musculus*), primarily a non-HPS clinical presentation is expected, which may be present along with other endemic infectious diseases in Barbados which share similar clinical symptomology. Thus, we present a report of the epidemiological features of human orthohantavirus infections, serological and molecular evidence of orthohantavirus infections along with *Leptospira* and multiple arboviral infections (DENV, ZIKV, and CHIKV) and serotyping attempts of orthohantavirus infections in Barbados. These should provide useful data to aid in the understanding, awareness, control and prevention of orthohantavirus infections in Barbados and the wider Caribbean.

## 2. Results

### 2.1. Laboratory Testing and Clinical Symptomology of Orthohantavirus-Positive Patients

**Figure 1:**
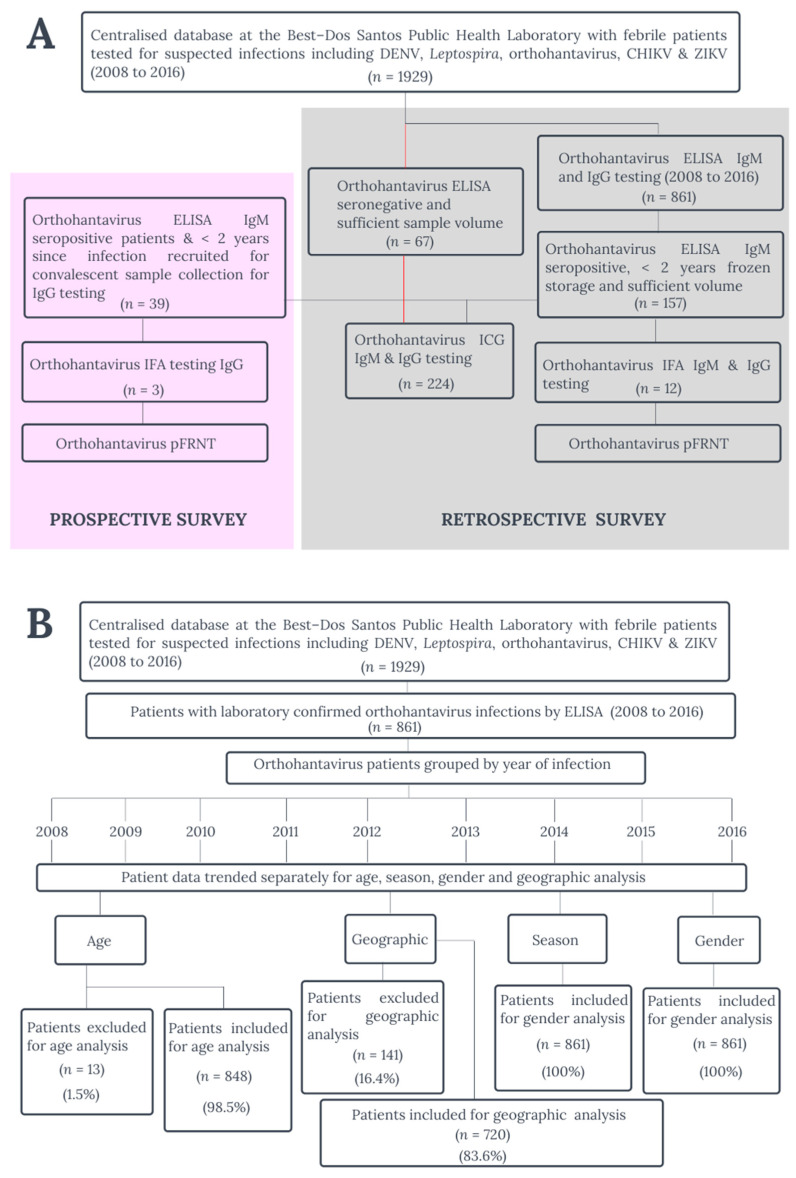
(**A**) Confirmatory serological testing methodology for the retrospective and prospective orthohantavirus survey studies, Barbados, 2008 to 2016. (**B**) Sampling and analysis methodology of the orthohantavirus epidemiology study, Barbados, 2008 to 2016.

To provide updated data on human orthohantavirus epidemiology in Barbados, two serosurveys were conducted in this study, namely (a) a retrospective serosurvey study using archived acute sera (orthohantavirus IgM- and immunoglobulin G (IgG)-seropositive) and (b) a prospective serosurvey study (<two years since enzyme linked immunosorbent assay (ELISA) IgM-seropositive result) where patients were recruited to obtain a convalescent serum sample for orthohantavirus IgG analysis (Figure 1A). Using a centralized database at Best-dos Santos Public Health Laboratory, St. Michael, Barbados, febrile patients (*n =* 1929) tested for suspected infections including DENV, *Leptospira*, CHIKV, ZIKV and orthohantavirus between 2008 and 2016 were identified (Figure 1A). A total of 1929 patients were screened for the presence of orthohantavirus-specific antibodies using orthohantavirus-specific ELISA IgM and IgG tests and 44.6% (861/1929) of the patient samples tested positive (Figure 1A). All the 861 orthohantavirus patient samples were ELISA IgM-positive and 132/861 (15.3%) were ELISA IgG-positive (Table 1).

Evidence of other pathogenic infections were observed among 25.6% (220/861) of orthohantavirus patients including DENV, ZIKV, CHIKV and *Leptospira* infections using NS1, RT-PCR and MAT assays as febrile patient sera tested were also under investigation for 24.7% DENV (213/861), 0.2% ZIKV (2/861), 0.2% CHIKV (2/861) and 0.4% *Leptospira* (3/861) infections (Figure 1A and Table 1). Laboratory testing for one patient revealed molecular evidence of ZIKV and DENV infection along with serological evidence of acute orthohantavirus infection representing the first case of multiple pathogenic infections including orthohantavirus in the Caribbean. From 2008 to 2016, a total of 861 orthohantavirus-specific ELISA IgM-positive patients who sought medical attention, including 297 hospitalized patients, were reported in Barbados (Table 1 and Figure 1B).

Clinical presentation among orthohantavirus patients is consistent with non-HPS (Table 2). The most common symptoms observed among orthohantavirus patients were headache (80.3%), fever (56.1%), joint pain (47.7%), gastrointestinal-related symptoms (vomiting, diarrhea and abdominal pain) (41.0%), eye (38.3%) and muscle pain (30%) (Table 2) [26]. Respiratory symptoms and cough were observed in 19.9% of orthohantavirus patients and could be due to orthohantavirus infection or other infections such as influenza, mammarenavirus and or other respiratory viruses (Table 2) [27,28,29].

### 2.2. Confirmatory Human Orthohantavirus ELISA, ICG, IFA and pFRNT Assay Results (Acute and Convalescent)

Confirmatory testing of ELISA-seropositive sera was done to establish the veracity of the ELISA results and to serotype the existing orthohantavirus strains in Barbados. From the 861 orthohantavirus-seropositive patients, 18.2% (157/861) met the criteria for confirmatory testing which entailed seropositive IgM results and sufficient sample volume (Figure 1A). Using immunofluorescence assay (IFA) testing, 9 (5.7%) of 157 acute patient sera (ELISA-positive using > 2.0 OD ratio cut-off) and 1 (1.6%) out of 67 ELISA-negative sera were IFA-positive (Table 3). These IFA-positive sera exhibited frequent seroreactivity to PUUV (7/9, 77.8%) and to a lesser extent SEOV (6/9, 66.7%) and none to HTNV (Table 3). The intensity of IFA-positive sera was highest for PUUV strain (Table 3). Only 1 ELISA-negative patient sample (H129) was IFA-positive (Table 3). Among convalescent patient sera tested only 3 (7.5%) of 39 recruited orthohantavirus ELISA-positive patients were IFA-positive, and only for PHV and not PUUV, SEOV or HTNV (Table 3). ELISA-negative with the confirmatory assays refers to patient sera with < 2.0 OD and not the manufacturer’s recommended < 1.1 OD ratio.

Immunochromatographic (ICG) testing revealed varied seroreactivity with known orthohantavirus strains (Table 3). ICG was conducted on 227 acute patient sera (157 seropositive and 67 seronegative by orthohantavirus-specific ELISA IgM testing) not convalescent sera (Figure 1A). Seropositive and seronegative samples were selected based on the criteria of less than two years of frozen storage and sufficient sample volume (Figure 1A). A total of 66 out of 67 (66/67, 98.5%) ELISA-negative (< 2.0 OD) patient sera were ICG-positive, yielding positive reactions to each of the PUUV (group B), DOBV and HTNV (group A) antigens. All ELISA-positive patient sera (157/157, 100%) and all IFA-positive patient sera (10/10, 100%) were ICG-positive exhibiting seroreactivity only to each of the PUUV, DOBV and HTNV antigens with notably no seroreactivity was unexpectedly observed for SEOV antigen (Table 3). Only 2 out of 10 (20.0%) IFA-positive acute sera were seroreactive to both SNV and ANDV antigens (Table 3). One ELISA-positive/IFA-negative patient sample was also positive to PUUV, SNV and HTNV by ICG strips (Table 3).

The serotyping of orthohantavirus strain(s) in Barbados was not successful using the pseudotype focus reduction neutralization test (pFRNT), since no IFA-positive acute patient sera (0/12) or IFA-positive convalescent sera (0/3) showed virus neutralization of known orthohantavirus strains (PUUV, SEOV, HTN, ANDV) above the threshold of 80%, indicating that none of these standard orthohantavirus strains were circulating in patients (Figure 1A and Table 3). 

A very strong seroreactivity to PUUV using ICG was observed with IFA-negative but ELISA-positive patient sera (Table 4). These patients exhibited a range of clinical presentations including those similar to non-HPS (fever, muscle and joint pain, hematuria, etc.) and includes three patients presenting with respiratory symptoms, difficulty breathing and requiring medical intensive care (Table 4). One of these patients was a bricklayer who originated from the United Kingdom but was a resident in Barbados (Table 4). Though this patient likely contracted this orthohantavirus infection in Barbados, it is not impossible for travelers from orthohantavirus endemic areas with active infections to travel to Barbados and present clinically.

### 2.3. Comparison of Sensitivity and Specificity of ELISA OD Ratios with IFA Testing

In comparison to IFA, ELISA assays exhibited a sensitivity $\left[\frac{true\;positives}{true\;positives+false\;negatives}\times 100\right]$ of 90% $\left[\frac{9}{\left(9+1\right)}\times 100\right]$ and a specificity $\left[\frac{true\;negatives}{true\;negative+false\;positives}\times 100\right]$ of 28.5% $\left[\frac{61}{\left(153+61\right)}\times 100\right]$ using the optical density (OD) ratio threshold of > 2.0 for ELISA positivity. For an OD ratio threshold of > 2.0, the positive likelihood ratio (PLR) $\left[\frac{sensitivity}{1-specificity}\right]$ was 1.26 $\left[\frac{0.9}{\left(1-0.285\right)}\right]$ (95% CI, 0.93 to 1.72) and the negative likelihood ratio (NLR) $\left[\frac{1-specificity}{sensitivity}\right]$ was 0.79 $\left[\frac{1-0.285}{\left(0.9\right)}\right]$ (95% CI, 0.58 to 1.08).

In comparison to IFA, ELISA assays with an OD cut-off of > 1.1 exhibited a sensitivity of 90% $\left[\frac{9}{10}\times 100\right]$ and a specificity of 25.2%$\;\left[\frac{54}{\left(160+54\right)}\times 100\right]$. For an OD ratio threshold of > 1.1, the PLR was 1.35 $\left[\frac{1}{1-0.252}\right]\;$(95% CI, 0.99 to 1.85), which is slightly higher than the PLR (OD ratio of > 2.0) and the NLR was 0.83 $\left[\frac{1-0.252}{0.9}\right]$ 95% CI, 0.61 to 1.14) slightly higher than the NLR (OD ratio of > 2.0) but the differences were not statistically significant. Therefore, the use of the manufacturer’s recommended OD threshold of 1.1 is valid and was used for epidemiological analysis. The 95% confidence intervals (CI) were calculated for each assay sensitivity and specificity using Microsoft Excel to determine the significance [30].

### 2.4. Orthohantavirus Outbreaks and Age Distribution 

**Figure 2:**
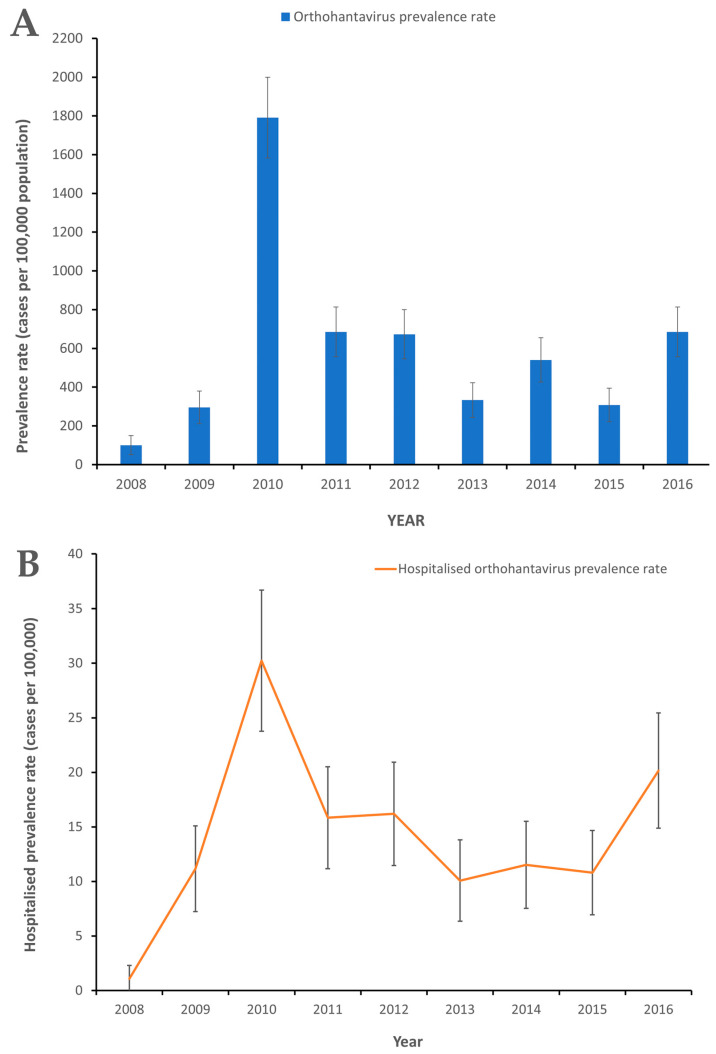
(**A**) Orthohantavirus prevalence rates among patients seeking medical attention in Barbados, 2008 to 2016. (**B**) Hospitalized orthohantavirus prevalence rates among patients seeking medical attention in Barbados, 2008 to 2016. The 95% confidence intervals (CI) were calculated to determine level of significance of differences in orthohantavirus prevalence rates.

Two major orthohantavirus outbreaks occurred in 2010 and 2016 with more hospitalizations occurring in 2010 than in 2016 (Figure 2A,B). A significantly higher orthohantavirus prevalence rate occurred in 2010 compared to 2009 with 99.7 (95% CI, 84.3 to 115.3) vs. 16.2 (95% CI, 10.0 to 22.4) cases per 100,000 population, respectively, and in 2016 compared to 2015 with 41.0 (95% CI, 31.1 to 50.0) and 17.6 (95% CI, 11.1 to 24.1) cases per 100,000 population, respectively (Figure 2A). The crude orthohantavirus prevalence rates varied by year from 5.8 (95% CI, 2.0 to 9.5) to 99.7 (95% CI, 84.3 to 115.3) cases per 100,000 population among patients with febrile illness who sought medical attention (Figure 2A). 

A significantly higher hospitalized orthohantavirus prevalence rate was observed during 2010, 30.2 (95% CI, 23.8 to 36.7) cases per 100,000 population than all other years in the study, except for 2016, with 20.2 (95% CI, 14.9 to 25.4) cases per 100,000 population (Figure 2B). The 95% CIs were calculated for each orthohantavirus prevalence rate using Microsoft Excel to determine the significant difference between orthohantavirus prevalence rates [30].

**Figure 3:**
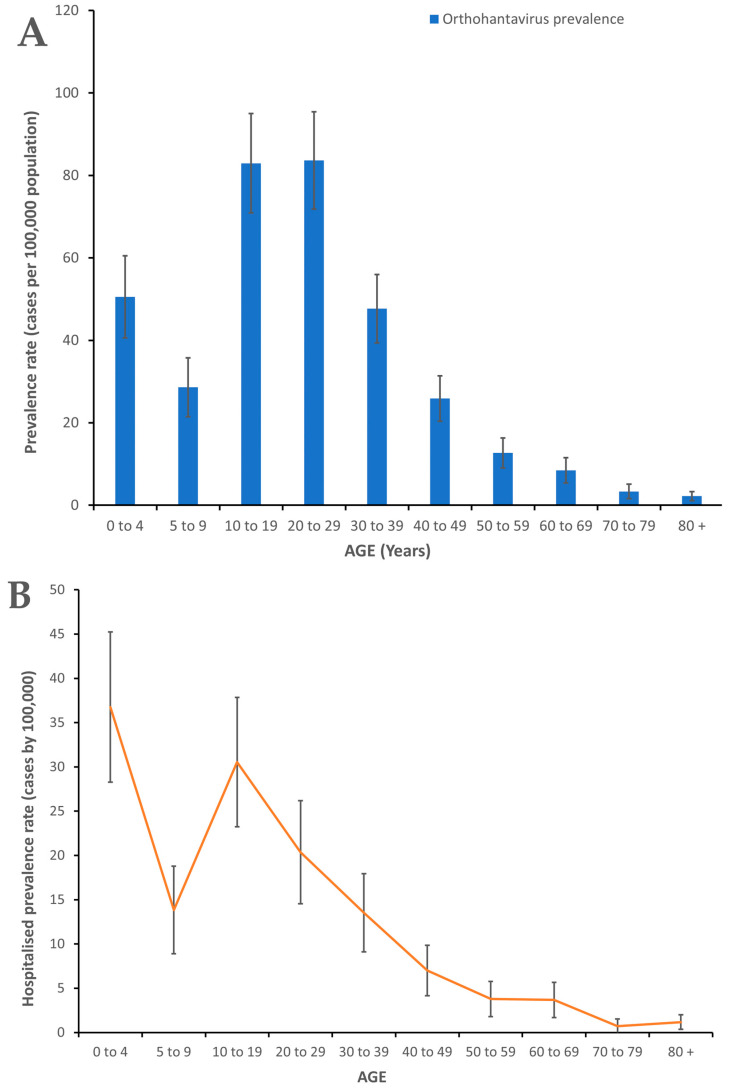
(**A**) Age-specific orthohantavirus prevalence rates among patients seeking medical attention in Barbados, 2008 to 2016. (**B**) Age-specific hospitalized orthohantavirus prevalence rates of among patients seeking medical attention in Barbados, 2008 to 2016. The 95% confidence intervals (CI) were calculated to determine level of significance of differences in orthohantavirus prevalence rates.

The highest orthohantavirus prevalence rates were observed with the 10 to 19 years age group, with 83.6 (95% CI, 71.8 to 95.4) cases per 100,000 population and the 20 to 29 years age group, 83.0 (95% CI, 70.9 to 95.0) cases per 100,000 population, which were both significantly higher than all the other age groups (Figure 3A). However, orthohantavirus hospitalization prevalence rates were highest among the 0 to 4 years age group, 36.8 (95% CI, 28.3 to 45.2) cases per 100,000 population, and was significantly higher than all age groups except the 10 to 19 age group (Figure 3B).

### 2.5. Season, Gender and Geographic Distribution of Human Orthohantavirus Infections

**Figure 4:**
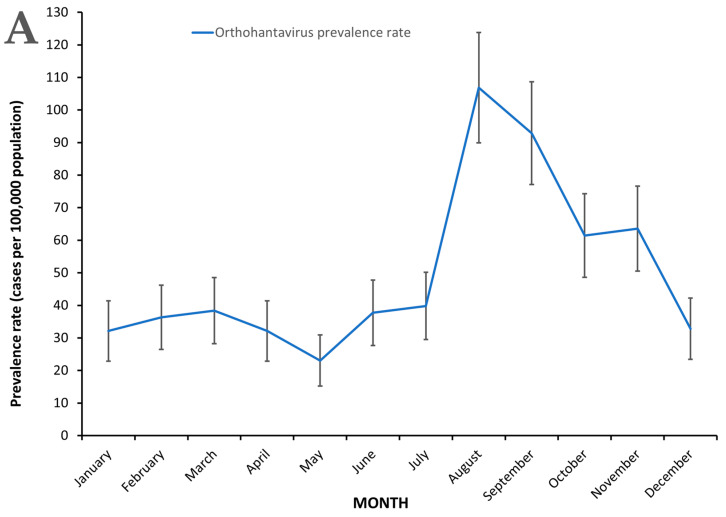

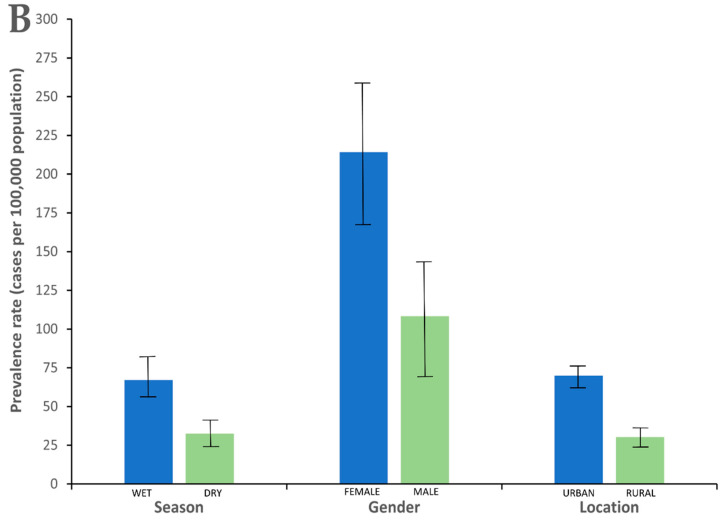
(**A**) Monthly orthohantavirus prevalence rates among patients seeking medical attention in Barbados, 2008 to 2016. (**B**) Comparison of mean orthohantavirus prevalence rates related to season, gender and location among patients seeking medical attention in Barbados, 2008 to 2016. Wet season in Barbados includes the months of June to November and the dry season December to May. Rural parishes include St. Lucy, St. Peter, St. John, St. Thomas, St. George, St. Andrew and St. Joseph and urban parishes include St. Michael, Christ Church, St. James and St. Philip. Statistically significant differences were observed with orthohantavirus prevalence rates between wet seasons vs. dry seasons, females vs. males, and between patients living in urban vs. rural parishes. The 95% confidence intervals (CI) were calculated to determine level of significance of the differences in orthohantavirus prevalence rates.

In Barbados, orthohantavirus infections occurred year-round but peaked in the months of August and September during the study period, 2008 to 2016 (Figure 4A). The monthly orthohantavirus prevalence rate among patients seeking medical attention ranged from 11.9 (95% CI, 6.5 to 17.2) cases per 100,000 population to 55.1 (95% CI, 43.6 to 66.6) cases per 100,000 population (Figure 4A). The highest prevalence rates were observed during August and September, 55.1 (95% CI, 43.6 to 66.6) and 47.9 (95% CI, 37.2 to 58.6) (Figure 4A) which were significantly higher than all other months during the study period, 2008 to 2016. 

Significantly more orthohantavirus infections occurred during the wet season than during the dry season (Figure 4B). The orthohantavirus prevalence rate was higher during the wet (rainy) season (June to November), 34.6 (95% CI, 25.5 to 43.7) cases per 100,000 population, than the dry season (December to May), 16.7 (95% CI, 10.4 to 23.1) cases per 100,000 population and this difference was statistically significant (Figure 4B). A total of 855 out of 861 (98.8%) orthantavirus patients were used in the seasonal analysis and orthohantavirus infections occurred year-round in Barbados (Figure 1B and Figure 4A). 

Female febrile patients were more likely to be infected with orthohantaviruses than males during the study period 2008 to 2016 (Figure 4B). The mean gender-specific orthohantavirus prevalence rates of patients seeking medical attention were significantly higher in females, 214.3 (95% CI, 161.8 to 266.7) cases per 100,000 population than males 108.4 (95% CI, 71.4 to 145.3) cases per 100,000 population (Figure 4B) and the male:female orthohantavirus infection ratio was approximately 1:2. The highest orthohantavirus prevalence rates among both males, 331.7 (95% CI, 267.0 to 396.3) cases per 100,000 population, and females, 616.0 (95% CI, 527.0 to 705.0) cases per 100,000 population, were observed in 2010 and were significantly higher than all other years in the study (data not shown). 

Urban parishes experienced higher orthohantavirus prevalence rates than rural parishes in Barbados among febrile patients seeking medical attention (Figure 4B). Febrile patients in urban parishes, 69.9 (95% CI, 63.4 to 76.4) cases per 100,000 population, were more likely to have orthohantavirus infections than those in rural parishes, 30.3 (95% CI, 25.3 to 35.2) cases per 100,000 population, during the study period of 2008 to 2016 (Figure 4B). Rural parishes include St. Lucy, St. Peter, St. John, St. Thomas, St. George, St. Andrew and St. Joseph and urban parishes include St. Michael, Christ Church, St. James and St. Philip.

## 3. Discussion

Orthohantavirus epidemiology studies in the Caribbean region countries have been sparse and we present the first population-wide orthohantavirus epidemiology study in both Barbados and the Caribbean. This study is useful in providing additional simultaneous pathogen infections of DENV, CHIKV, ZIKV and *Leptospira* in Barbados. 

### 3.1. Clinical Symptoms

Headache and fever were the two most frequently observed symptoms among orthohantavirus cases and are consistent with observations from orthohantavirus infections in Europe and North America [31,32]. Severe gastrointestinal (GI) symptoms including abdominal pain, nausea and vomiting were previously found to be common in patients with PUUV infection and co-circulation of *Leptospira*, ZIKV, CHIKV and DENV exists in Barbados [33,34,35,36]. Thus, the high frequency of gastrointestinal-related symptoms, joint and muscle pain among orthohantavirus patients in Barbados is not unexpected, as this has been previously observed in Indonesia [31,32,37]. Simultaneous or previous DENV, ZIKV and or CHIKV infections can lead to rash and chronic arthralgia lasting several months after infection [37,38,39,40,41]. This potentially highlights the need for orthohantavirus studies in regions other than those traditionally reporting orthohantavirus to expand knowledge of orthohantavirus clinical presentations and its epidemiology in different geographical, pathogen endemicity and host genetic contexts. Typically, a non-HPS clinical presentation was observed during this study; however, the observance of respiratory symptoms and the need for medical intensive care indicate possible rare clinical complications in Barbados [27,28,29]. The potential also exists for travelers from orthohantavirus-endemic regions to become imported cases upon travelling to Barbados given the long incubation period of infection (8 to 45 days) [42,43,44,45].

### 3.2. Orthohavirus Outbreaks, Age and Gender

Orthohantavirus outbreaks occurred in Barbados during 2010 and 2016 and represent the first report of orthohantavirus outbreaks in the English-speaking Caribbean. The mean annual seroprevalence rate observed in this study among patients that presented with febrile illness and sought medical care was higher than that reported from other countries including Brazil (1.0 cases/100,000 population), USA (0.009 cases/100,000 population), Chile (0.29 cases/100,000 population) even China (1.5 cases/100,000 population) [38,39,40]. This may be due to a disparity in research in the Americas for HPS-related infections by New-World orthohantaviruses compared to non-HPS-related orthohantavirus infection caused by SEOV [46,47]. In areas where non-HPS orthohantavirus disease was examined, e.g., China (28.62 cases/100,000 population), a higher seroprevalence rate was observed [38]. In areas where the circulating or endemic strain(s) is unknown, ELISA assays may initially be better to detect orthohantavirus cases. The highest orthohantavirus prevalence rates among patients with fever illness that sought medical care were observed in persons zero to four years of age, which agrees with a previous study of children in Barbados [20]. In 2002, the seroprevalence rates among patients was less than in our current study and the difference could be attributed to the statistically significant difference in sample sizes of both studies; the 2002 study examined 75 patients, whereas the current study examined 861 patients. 

Approximately 150,000 to 200,000 patients with HFRS are hospitalized each year around the world [48]. Severe HFRS cases do occur, resulting in hospitalization and the risk factors include pre-existing co-morbidities, home proximity to heavily vegetated/wooded areas, virus strain, gender, smoking and age [49,50,51,52]. A higher orthohantavirus case fatality rate among females than males has been previously observed with HPS, HFRS and NE but this was not so for this study [53,54,55]. A single death occurred among orthohantavirus cases, which was a 65-year-old male, admitted to the high-dependency unit during hospitalization, exhibiting respiratory symptoms, fever, rash and joint pains. The patient was orthohantavirus IFA-negative but ELISA- and ICG strip-positive. However, orthohantavirus was not identified as the cause of death. Hospitalization rates were highest among the 0 to 4 years age group and this may reflect the clinical perspective of acute infections in very young children. Physicians are more likely to hospitalize young babies and toddlers to monitor their clinical progression during febrile illness as such illnesses can be more life threatening in early age.

Sex bias does occur in infectious disease epidemiology including orthohantavirus infections [24,27]. In Barbados, females were more likely to be infected with orthohantavirus than males and this agrees with studies in the Netherlands and Brazil [56,57]. More research is needed to provide more insight into this gender disparity with respect to orthohantavirus infection in Barbados and possibly the Caribbean.

### 3.3. Influence of Seasonality and Geographic Location

Orthohantavirus transmission is influenced by environmental and climatic factors including rainfall, topography and vegetation [58,59,60]. Orthohantavirus outbreaks were observed during 2010 and 2016 in Barbados and these could be due to enhanced surveillance due to ongoing DENV epidemics and thus greater awareness among physicians for persons presenting with dengue-like symptoms [61,62]. High rainfall was associated with increased orthohantavirus transmission in Barbados as higher infection rates were observed during the wet season compared to the dry season. Rainfall can permit moist soil, which facilitates rodent burrowing, breeding, survival and the proliferation of vegetation and food for rodents [59,60]. Conversely, excessive rainfall and or extreme weather events including flooding can result in the reduction of rodent population, reduced risk of orthohantavirus transmission and reduced orthohantavirus seroprevalence [63]. Other climatic factors influencing orthohantavirus transmission include atmospheric moisture variability and temperature [63], so tropical climatic conditions such as high temperature and humidity in Barbados could influence the survival of orthohantaviruses in the environment and their transmission. 

Urbanization can contribute to the generation of more waste with higher population density and an increased proliferation of rodents. This appears to influence orthohantavirus infection in Barbados with higher infection rates observed in patients from urban rather than rural areas [64,65]. This was also observed in Brazil, where females were more likely to become infected and occupation (housewives) was identified as a possible risk [57]. However some research has shown rural areas with a higher risk of orthohantavirus infection than urban areas [65]. More research on the influence of abiotic factors in orthohantavirus transmission is therefore necessary to understand the orthohantavirus ecology in Barbados and Caribbean. 

### 3.4. Strengths

Several key findings were observed in this study including (1) the first report of orthohantavirus outbreaks during 2010 and 2016 in the Caribbean; (2) the first report of serological evidence of simultaneous multiple pathogen infections including *Leptospira*, ZIKV, DENV and CHIV with orthohantavirus infections; (3) an unusually higher orthohantavirus prevalence rate among female febrile patients than males; (4) generally mild and atypical clinical symptoms not traditionally observed in orthohantavirus endemic regions consistent with non-HPS clinical presentation; (5) year-round orthohantavirus transmission with a seasonal peak in August and generally higher in the wet season; and (6) higher prevalence rates in urban vs. rural parishes.

### 3.5. Study Limitations

Every study can benefit from improvements, and this study is no exception. Study improvements include sampling a wider proportion of the population other than febrile persons seeking medical attention [66,67,68]. In addition, the IgM seropositivity ideally should be accompanied with another sample two or three weeks later, but paired sera were rarely submitted for follow-up testing as patients seldom return for testing once they have recovered.

### 3.6. Recommendations

In an effort to improve biosecurity in Barbados and the Caribbean region, some possible recommendations could include (1) enhanced biosecurity surveillance at ports of entry to minimize the risk of the introduction of imported orthohantavirus cases and also alien rodent species into Barbados due to regional trade and transport, (2) enhanced public awareness to inform persons of transmission risk and peak periods for orthohantavirus infection, (3) the use of a cross-sectional study carried out with asymptomatic volunteers from various communities across the island to determine true orthohantavirus prevalence, and (4) qualitative public health research to elucidate the reason(s) for this disparity between sexes and urban and rural areas as possible risk factors of orthohantavirus infection. 

### 3.7. Conclusions

Orthohantavirus infections are still occurring in Barbados and in some patients along with multiple pathogen infections (CHIKV, ZIKV, DENV and *Leptospira*). Orthohantavirus infections are more prevalent during periods of high rainfall (rainy season) with peak transmission in August, females are more likely to be infected than males and orthohantavirus infections are more likely among patients from urban rather than rural parishes in Barbados.

## 4. Materials and Methods

### 4.1. Ethical Approval

The study (IRB No. 181110–A) was approved by the Institutional Review Board (IRB) on Ethics in Research on Human Subjects at The University of the West Indies (UWI), Cave Hill, St. Michael, Barbados combined with the Ministry of Health on 11 July 2013 and the Ethics Committee at the Queen Elizabeth Hospital (QEH), Martindale’s Road, St. Michael, Barbados on 19 August 2013 prior to the start of data collection and analyses. Informed consent was not necessary for retrospective acute febrile patient sera, but informed consent was obtained for the collection of convalescent sera.

### 4.2. National Human Surveillance Program

In Barbados, all suspected febrile patients from local hospitals and local doctors are referred to the Best-dos Santos Public Health Laboratory based on clinical symptoms consistent with DENV, CHIKV, ZIKV, *Leptospira* and orthohantavirus infections, which are characterized by fever, malaise, myalgia, arthralgia, rash, retro-orbital pain, abdominal pain, nausea and vomiting. Convenience sampling of patients from this database then permits a good representation of the entire population in Barbados with febrile illness (Figure 1B). All probable cases of orthohantavirus infections are submitted to a central Best-dos Santos Public Health Laboratory, where sera are archived at −20 °C. 

### 4.3. Case Definition

Orthohantavirus cases were confirmed by detection of orthohantavirus-specific IgM and IgG in patients’ serum (samples within 5 to 15 days of illness) with an orthohantavirus IgM and IgG enzyme-linked immunosorbent assay (ELISA) kit, based on the manufacturer’s instructions (Focus Diagnostics, Cypress, CA, USA) [19] (Figure 1A). The Focus Diagnostics Hantavirus DxSelect™ kit (Focus Diagnostics, Cypress, CA, USA) uses a cocktail of baculovirus-derived recombinant nucleoprotein (rNP) of orthohantavirus species. Using an rNP cocktail allows for detecting antibodies to a broad range of orthohantavirus variants, including antibodies to the most clinically relevant pathogenic strains of orthohantaviruses, i.e., SEOV, HTNV, PUUV, DOBV, and SNV. We defined the case of a clinical laboratory orthohantavirus infection according to the Centers for Disease Control and Prevention hantavirus case definition for non-HPS, specifically “the detection of hantavirus-specific immunoglobulin (Ig) M” [69].

### 4.4. Study Design and Sampling

Using centralized database at Best-dos Santos Public Health Laboratory, St. Michael, Barbados, febrile patients (*n =* 1929) tested for suspected infections including DENV, *Leptospira*, CHIKV, ZIKV and orthohantavirus between 2008 and 2016 were identified (Figure 1A).

For ELISA testing, the use of >1.1 OD cut-off has been previously questioned with a preference for a more stringent cutoff of >2.0 being advanced to avoid the high number of false positives [70,71]. From the orthohantavirus-seropositive patients (*n* = 861), samples were selected for confirmatory testing (*n* = 157) using criteria for further confirmatory testing which entailed having orthohantavirus-seropositive IgM test results, less than two years of frozen storage and sufficient available sample volume (Figure 1A).

For confirmatory orthohantavirus testing, two separate orthohantavirus patient studies were conducted; first, a retrospective survey to confirm orthohantavirus infection with 157 archived acute sera (with ELISA OD ratio > 2.0) and 67 archived acute sera (with ELISA OD ratio < 2.0) selected from a list of orthohantavirus ELISA IgM-positive patients from 2008 to 2018 was conducted with IFA, ICG and pFRNT tests (Figure 1A). The 67 seronegative samples were selected based on available sample volume, <two years sample storage and seronegative orthohantavirus ELISA test (Figure 1A). Second, a prospective study with convalescent sera collected from 39 recruited previous orthohantavirus ELISA IgM-positive patients conducted in 2019 were screened with IFA, ICG and pFRNT tests. The criteria for selection of recruited patients were a seropositive orthohantavirus ELISA IgM test and <two years since last seropositive orthohantavirus IgM result.

A list of orthohantavirus ELISA IgM-positive patients (*n =* 861), was produced based on ELISA serological tests (Figure 1B). The list of orthohantavirus cases was sorted and grouped by the year of orthohantavirus disease/symptom onset 2008 to 2016. For each year, the cases were analyzed by age, gender, geographical location and year (Figure 1B). Within these epidemiological categories, seroprevalence and hospitalization rates (per 100,000 population) were calculated using the Barbados 2010 census data as the denominator. Age standardization was carried out using the World Health Organization (WHO) standard [72]. For geographic and gender analysis, parish (geographical region in Barbados), male and female populations from the Barbados 2010 national census were used, as the denominator, to calculate the respective seroprevalence rates. Outbreaks were defined as a significant difference between prevalence rates in the preceding and outbreak year. The 95% CIs were calculated for each orthohantavirus prevalence rate using Microsoft Excel to determine the significant difference between orthohantavirus prevalence rates [30]. For human orthohantavirus cases, the frequencies of relevant clinical symptoms were calculated. 

### 4.5. Serological Testing of Human Sera

Sera samples from febrile patients suspected of acute DENV infection (*n* = 1929) were routinely screened by ELISA for orthohantavirus-specific IgM and IgG (Hantavirus IgM and IgG Focus Dx, Cypress, CA, USA) (Figure 1A) [19]. Orthohantavirus IgM-seropositive patient sera (*n* = 861) which met the criteria of being orthohantavirus IgM-seropositive, less than two years frozen storage and having sufficient available sample volume (*n* = 157) were subjected to confirmatory testing by IFA (PUUV, SEOV, PHV) at the University of Helsinki, Finland), ICG strips (Global Hantavirus Euroimmun, Luebeck, Germany) [73] and species typing was performed using pFRNT against PUUV, SEOV, HTNV and ANDV viruses using the relevant positive and negative controls [74] (Figure 1A). For IFA-positive acute human sera (*n* = 12), pFRNT assays were conducted as previously described using PUUV, DOBV, ANDV, HTNV strains to confirm orthohantavirus infection with the end-point titer calculated as the quantity of neutralizing antibody that resulted in an 80% reduction in fluorescent foci using the relevant positive and negative controls [74] (Figure 1A).

## Figures and Tables

**Table 1:** Laboratory testing results of orthohantavirus-positive patients for other pathogenic infections in Barbados, 2008 to 2016.

Year	Samples Tested	Orthohantavirus Positive *	Orthohantavirus ELISA		DENV			ZIKV rRT–PCR	CHIKV rRT–PCR	*Leptospira*
		(%)	IgM +	IgG +	IgM +	NS1	rRT–PCR			
2008	749	2.1	16	5	4	0	0	0	0	0
2009	472	10.2	48	4	6	0	0	0	0	0
2010	2033	13.7	279	1	71	3	2	0	0	2
2011	966	11.3	109	19	19	1	0	0	0	1
2012	1649	6.5	107	33	26	2	2	0	0	0
2013	2758	1.9	53	15	19	1	2	0	0	0
2014	2529	3.4	86	29	8	0	0	0	2	0
2015	327	15.0	49	12	10	1	0	0	0	0
2016	696	16.4	114	14	34	0	2	2	0	0
TOTAL	1929		861	132	197	8	8	2	2	3

**Key:** * All orthohantavirus cases were enzyme-linked immunosorbent assay (ELISA) immunoglobulin M (IgM) positive and some were both orthohantavirus IgM and immunoglobulin G (IgG) positive. Orthohantavirus-positive patients were also tested for dengue virus (DENV), Zika virus (ZIKV), Chikungunya virus (CHIKV) and *Leptospira* infection. DENV infection was confirmed by DENV-specific ELISA IgM, DENV non-specific protein 1 (NS1) antigen test and real-time reverse transcriptase (rRT–PCR). ZIKV and CHIKV infection were confirmed using ZIKV- or CHIKV-specific rRT–PCR respectively and *Leptospira* infection was confirmed using microagglutination (MAT) titers.

**Table 2:** Frequency of clinical symptoms in orthohantavirus-positive patients in Barbados (2008 to 2016).

Symptoms	No. of Patients	Frequency (%)
Fever	691	80.3
Headache	483	56.1
Joint pain	411	47.7
Gastrointestinal-related symptoms *	353	41
Eye pain	330	38.3
Muscle pain	258	30
Anorexia	138	16
Rash	132	15.3
Respiratory symptoms	132	15.3
Jaundice	71	8.2
Cough	40	4.6
Lethargy	36	4.2
Thrombocytopenia	30	3.5
Bleeding	22	2.6
Hematuria	20	2.3
Renal complications	7	0.8

* Gastrointestinal-related symptoms include vomiting, abdominal pain and diarrhea.

**Table 3:** Confirmatory orthohantavirus laboratory test results on acute and convalescent human serum samples.

Clinical Status	Patient ID	ELISA		IFA, > 20				ICG Strip, Intensity Rating						pFRNT, Reciprocal Endpoint Titer			
		OD Ratio > 2.0		Group B	Group A			Group B	Group A			Group C		Group B	Group A		Group C
		IgM	IgG	PUUV	SEOV	HTNV		PUUV	SEOV	HTNV	DOBV	SNV	ANDV	PUUV	SEOV	HNTV	ANDV
Acute	H35	+	−	+	−	−		(+)	(+)	(+)	(+)	−	−	< 40	< 40	< 40	< 40
*n =* 157	H36	+	−	+	−	−		(+)	(+)	(+)	(+)	−	−	< 40	< 40	< 40	< 40
	H67	+	+	+++	+	−		++	−	++	++	−	−	< 40	< 40	< 40	< 40
	H72	−	+	−	+	−		(+)	−	(+)	(+)	−	−	< 40	< 40	< 40	< 40
	H76	−	+	+	+	−		+	−	+	+	−	−	< 40	< 40	< 40	< 40
	H90	+	−	++	+	−		+	−	+	+	−	−	< 40	< 40	< 40	< 40
	H111	+	−	−	+	−		+	−	+	+	−	−	< 40	< 40	< 40	< 40
	H112	+	−	+	−	−		+	−	+	+	−	−	< 40	< 40	< 40	< 40
	H114	+	−	−	+	−		+	−	+	+	−	−	< 40	< 40	< 40	< 40
	H123	+	−	−	−	−		+++	−	−	−	−	−	< 40	< 40	< 40	< 40
	H128	+	−	−	−	−		+	−	−	−	−	−	< 40	< 40	< 40	< 40
	H129 *	−	−	+	+	−		++	−	−	−	−	−	< 40	<40	< 40	< 40
		**ELISA**, **Acute (>2.0 OD ratio)**		**IgM IFA, Acute Sample**				**IgG IFA, Convalescent**									
	**Patient ID**	**IgM**	**IgG**	**PUUV**	**SEOV**	**HTNV**	**PHV**	**PUUV**	**SEOV**	**HTNV**	**PHV**						
Convalescent																	
(*n =* 39)	H2/C4	+	+	−	−	−	−	−	−	−	+						
3/39 or 7.5%	H50/C15	+		−	−	−	−	−	−	−	+						
	H116/C28	+		−	−	−	−	−	−	−	+						

Group A orthohantavirus antigens include Seoul virus (SEOV), Hantaan virus (HTNV) and Dobrava virus (DOBV), group B orthohantavirus antigens Puumala (PUUV) and Prospect Hill virus (PHV) and group C orthohantavirus antigens Sin Nombre virus (SNV) and Andes (ANDV). Laboratory testing included enzyme linked immunosorbent assay (ELISA), reverse transcriptase polymerase chain reaction (RT-PCR), immunofluorescent assay (IFA), immunochromatographic test (ICG) strips and pseudotype focus reduction neutralization test (pFRNT). Acute sera are indicated by the ‘H’ and convalescent sera by ‘C’. **Key:** no band/fluorescence ornegative result (-); very weak fluorescence, borderline (+); medium to strong fluorescence, positive result (+, ++); very strong fluorescence comparable to control, strong positive (+++), patient sera with < 2.0 ELISA optical density (OD) ratio (*).

**Table 4:** Comparison of human sera seoreactivity using orthohantavirus-specific IFA and ICG strips.

	Sample	Age	Sex	IFA	ELISA	ICG STRIPS						Clinical Symptoms
						Group A			Group B	Group C		
						Murinae			Arvicolinae	Sigmodontinae		
						DOBV	SEOV	HTNV	PUUV	SNV	ANDV	
Human	H38	88	M	neg	pos	+	–	+	+++	–	–	Jaundice, anorexia, hematuria and muscle pain.
	H67	15	F	pos	pos	+++	–	+++	+++	–	–	Fever, rash, joint pain and heartburn but was not hospitalized and the patient was a resident Barbadian.
	H29	65	M	neg	pos	++	+	+++	+++	+	+	Fever, rash, joint pain and respiratory symptoms, was admitted to the high dependency unit at the QEH hospital and later died.
	H22	24	M	neg	pos	–	–	–	+++	++	–	Respiratory symptoms and petechiae; admitted to the medical intensive care unit (MICU) at the QEH hospital. Bricklayer by profession, UK by origin but resident in Barbados.
	H37	8 months	M	neg	pos	+++	–	+++	+++	–	–	Fever, respiratory symptoms, difficulty breathing with rapid, weak pulse, and was admitted to the pediatric intensive care unit at the QEH hospital

Group A orthohantavirus antigens include Seoul virus (SEOV), Hantaan virus (HTNV) and Dobrava virus (DOBV), group B orthohantavirus antigens Puumala (PUUV) and Prospect Hill virus (PHV) and group C orthohantavirus antigens Sin Nombre virus (SNV) and Andes (ANDV). Laboratory testing included enzyme linked immunosorbent assay (ELISA), reverse transcriptase polymerase chain reaction (RT-PCR), immunofluorescent assay (IFA), immunochromatographic test (ICG) strips and pseudotype focus reduction neutralization assay (pFRNT). Acute sera are indicated by the ‘H’ and convalescent sera by ‘C’. ‘Neg’ is for negative and “Pos” is for positive. Samples with the designation ‘H” are acute human sera with ELISA-positive results. **Key:** no band/fluorescence or negative result (–); very weak fluorescence, borderline (+); medium to strong fluorescence, positive result (+, ++); very strong fluorescence comparable to control, strong positive (+++).

## Data Availability

Not applicable.
